# Interleukin 33 supports squamous cell carcinoma growth via a dual effect on tumour proliferation, migration and invasion, and T cell activation

**DOI:** 10.1007/s00262-024-03676-8

**Published:** 2024-04-25

**Authors:** Graziela Perri, Vanessa Garcia Vilas Boas, Maria Renata Sales Nogueira, Edgard José Franco Mello Júnior, Ana Lucia Coelho, Edwin M. Posadas, Cory Hogaboam, Karen A Cavassani, Ana Paula Campanelli

**Affiliations:** 1 Department of Biological Sciences, Bauru School of Dentistry, University of São Paulo, Al. Dr. Octávio Pinheiro Brisolla, Bauru, SP 17012-901 Brazil; 2 Research and Teaching Division, State Department of Health, Instituto Lauro de Souza Lima, Bauru, SP Brazil; 3 Department of Medicine, Division of Pulmonary and Critical Care Medicine, Cedars-Sinai Medical Center, Los Angeles, CA 90048 USA; 4 Samuel Oschin Comprehensive Cancer Institute, Cedars-Sinai Medical Center, Los Angeles, CA 90048 USA

**Keywords:** IL-33, Squamous cell carcinoma, Proliferation, Motility, Invasion

## Abstract

Interleukin (IL)-33 is an important cytokine in the tumour microenvironment; it is known to promote the growth and metastasis of solid cancers, such as gastric, colorectal, ovarian and breast cancer. Our group demonstrated that the IL-33/ST2 pathway enhances the development of squamous cell carcinoma (SCC). Conversely, other researchers have reported that IL-33 inhibits tumour progression. In addition, the crosstalk between IL-33, cancer cells and immune cells in SCC remains unknown. The aim of this study was to investigate the effect of IL-33 on the biology of head and neck SCC lines and to evaluate the impact of IL-33 neutralisation on the T cell response in a preclinical model of SCC. First, we identified epithelial and peritumoural cells as a major local source of IL-33 in human SCC samples. Next, in vitro experiments demonstrated that the addition of IL-33 significantly increased the proliferative index, motility and invasiveness of SCC-25 cells, and downregulated *MYC* gene expression in SCC cell lines. Finally, IL-33 blockade significantly delayed SCC growth and led to a marked decrease in the severity of skin lesions. Importantly, anti-IL-33 monoclonal antibody therapy increase the percentage of CD4^+^IFNγ^+^ T cells and decreased CD4^+^ and CD8^+^ T cells secreting IL-4 in tumour-draining lymph nodes. Together, these data suggest that the IL-33/ST2 pathway may be involved in the crosstalk between the tumour and immune cells by modulating the phenotype of head and neck SCC and T cell activity. IL-33 neutralisation may offer a novel therapeutic strategy for SCC.

**Supplementary Information:**

The online version contains supplementary material available at 10.1007/s00262-024-03676-8.

## Introduction

Interleukin (IL)-33 is a pleiotropic cytokine that modulates the activities of tumour and immune cells [1]. IL-33 signals through the ST2 receptor, which exists in two forms, a soluble form (sST2), which acts as a decoy receptor, sequesters free IL-33 and does not signal, and a membrane bound form (ST2), which activates the MyD88/NF-κB signalling pathway to modulate immune cell functions [2].

Within the tumour microenvironment, epithelial cells, dendritic cells, macrophages, myeloid-derived suppressor cells (MDSCs), fibroblasts and cancer cells produce IL-33 [3]. However, the crosstalk between IL-33, cancer and immune cells in squamous cell carcinoma (SCC) remains unclear. IL-33 is known to play multiple and sometimes opposing roles in tumourigenesis [4]. It is a possible prognostic marker of cancer development that exerts a direct effect on tumourigenesis, proliferation and metastasis of tumour cells by remodelling the tumour microenvironment [5]. In addition, IL-33 drives chronic inflammation that supports tumour growth and promotes the activation of regulatory T cells (Tregs) and/or MDSCs [3, 6]. Conversely, IL-33 inhibits tumour growth by enhancing the activation of natural killer (NK) cells and cytotoxic CD8^+^ T cells [7–9], whereas mice lacking ST2 show attenuated SCC progression due to increased NK cell–mediated cytotoxicity [10]. Recently, it has been reported that the IL-33–transforming growth factor beta (TGFβ) signalling loop that connects tumour-initiating cells and FcERIα^+^ macrophages [11]. Moreover, it is now clear that IL-33 and cancer cell interactions within the tumour microenvironment regulate cancer stem cell properties and play an important role in tumour progression and metastasis [12].

In this study, we aimed to evaluate the role of IL-33 on the biology of the tumour cells and the T cell activation status during SCC. Analysis of human SCC biopsies showed the presence of IL-33 in epithelial cells as well as the tumour surroundings. These findings led us to evaluate the effect of IL-33 in the motility, proliferation and invasion of human SCC cells in vitro. We found that IL-33/ST2 signalling has a direct effect on the motility, invasion and proliferation SCC cells, and it is independent of tumour ‘plasticity’ genes, including neuroendocrine markers and genes that participate in epithelial-to-mesenchymal transition (EMT) and stemness. Interestingly, the expression of the transcription factor *MYC* was downregulated in the presence of IL-33 in SCC-25 and Detroit 562 cells, two head and neck squamous cell carcinoma (HNSCC) cell lines. However, IL-33 neutralisation in vivo led to a more quiescent tumour characterised by the infiltration of CD4^+^ T cells producing interferon gamma (IFNγ) (Th1-like) and decreased Th2-like cells (CD8^+^ and CD4^+^ T cells expressing IL-4) in the tumour microenvironment. These findings indicate that IL-33 plays a role in both tumour cell and immune activation. Targeting the IL-33/ST2 pathway may represent a potential immunotherapy for human SCC.

## Materials and methods

### SCC samples and healthy volunteers

To analyse IL-33 expression in SCC samples, we reviewed patients with carcinomas that were surgically resected and originally diagnosed with SCC at Instituto Lauro de Souza Lima between 2014 and 2016. There were tumour slides and blocks available for histological and immunohistochemical evaluation from 14 patients (median age 76.14 ± 10.25 years, range 16–99 years, 9 men and 5 women). This retrospective study was approved by the Institutional Review Board of Instituto Lauro de Souza Lima (37644714.7.0000.5475).

### Immunohistochemistry

Immunohistochemical staining was performed on formalin-fixed, paraffin-embedded tissue sections according to published protocols [12]. A human anti-IL-33 monoclonal antibody (clone O95760, R&D Systems, Minneapolis, MN, USA) was used to analyse IL-33. Briefly, the slides were deparaffinised, rehydrated and then rinsed in distilled water for 5 min. To block the endogenous peroxidase activity, the slides were incubated in 0.5% hydrogen peroxide in methanol. After washing, the slides were incubated with the primary anti-IL-33 antibody overnight at 4 °C. Next, the slides were washed with phosphate-buffered saline (PBS) and incubated with the appropriate biotinylated antibody for 1 h at room temperature. The staining was visualised using Impact DAB solution (Vector Laboratories). The slides were counterstained with haematoxylin.

### SCC cell lines

SCC-25 (CRL1628TM, human papilloma virus [HPV] negative) and Detroit 562 (HPV negative) cells were purchased from ATCC (Manassas, VA, USA). SCC25 cells were cultured in Dulbecco’s Modified Eagle Medium (DMEM)/F12 (GibcoBRL, Waltham, MA, USA) supplemented with hydrocortisone (400 ng/mL), 10% heat-inactivated foetal bovine serum (FBS) (Omega Scientific, Inc, Tarzana, CA, USA), 50 IU/mL penicillin and 50 µg/mL streptomycin (GibcoBRL). Detroit 562 cells were cultured in Eagle’s Minimum Essential Medium (EMEM) (GibcoBRL) with 10% FBS, 50 IU/mL penicillin and 50 µg/mL streptomycin (GibcoBRL) in 5% CO_2_ at 37 °C. The cells were negative for mycoplasma contamination (MycoAlert Mycoplasma Detection Kit, Lonza, Walkersville, MD, USA).

### Proliferation assay

For each SCC cell line, 1 × 10^4^ cells/well were seeded in 96-well plates and allowed to adhere at 37˚C and 5% CO_2_. After 24 h, the cells were fasted in 0.1% bovine serum albumin (BSA) for 4 h. Then, the cells were incubated with fresh complete medium in the presence of recombinant human IL-33 (rhIL-33, R&D Systems) at 0, 10, 50, and 100 ng/mL. The proliferation rate based on cell confluence was determined by live cell imaging (10× objective lens) using the IncuCyte ZOOM integrated software (Sartorius, Ann Arbor, MI, USA). At different time points, the software automatically calculated cell confluency. The y-axis values represent the fold change based on the baseline cell confluency without rhIL-33 at 0 h.

### Cell wound healing assay

The scratch assay was performed as described previously [13]. SCC cell lines were seeded in an Essen Imagelock 96-well plate (1.5 × 10^4^ cells/well) and maintained until reaching confluency. After serum starvation with 0.1% BSA, a wound was made in the cell monolayer using a 96-well wound-maker tool with polytetrafluoroethylene (PTFE) pin tips (ESSEN BioScience), according to the manufacturer’s instructions. The scratched wells were incubated with fresh starvation medium in the absence or presence of rhIL-33 (0, 10, 50 and 100 ng/mL) (R&D Systems) in 5% CO_2_ at 37 °C. Live-cell images were taken every 2 h for up to 48 h with the IncuCyte ZOOM system. The IncuCyte ZOOM integrated software was used to analyse the results.

### Cell invasion assay

SCC cell lines were fasted in 0.1% BSA and then plated (1 × 10^5^) on transwell inserts (8 μm) coated with Matrigel matrix, phenol red free (BD Biosciences, Franklin Lakes, NJ, USA). The inserts were placed in 24-well plates containing 500 µL of cell-free rhIL-33 (0, 10, 50 and 100 ng/ml) (R&D Systems), and incubated at 37 °C for 48 h. The cells attached to the bottom of the membrane were fixed with 4% paraformaldehyde and stained with 0.1% (v/v) crystal violet. The inserts were washed and photographed at 10× magnification using an inverted microscope (EVOS M5000, Invitrogen MA, USA). ImageJ (National Institutes of Health, Bethesda, MD, USA) was used to count the cells.

### Reverse transcription-quantitative polymerase chain reaction (RT-qPCR)

Total RNA was extracted from the SCC cell lines with TRIzol (Life Technologies, Invitrogen, Carlsbad, CA, USA). One microgram of total RNA was reverse transcribed to complementary DNA (cDNA), which was used for qPCR (run on an Applied Biosystems Viia 7 instrument; Thermo Fisher Scientific). Target gene expression was normalised to the expression of a housekeeping gene, 18 S or GAPDH. Relative gene expression was calculated by using standard 2^-∆∆Ct^ method. All primers for RT-qPCR were designed and synthesised by IDT Technologies (Coralville, IA, USA).

### Animal model of SCC

The Animal Care and Use Committee of the Bauru School of Dentistry, University of São Paulo, approved the animal procedures [012/2017]. All animals were maintained in compliance with the Guide for the Care and Use of Laboratory Animals prepared by CONCEA. Eight-week-old female BALB/c mice were obtained from the School of Medicine of São Paulo of Ribeirão Preto, University of São Paulo. The mice were subjected to a two-stage carcinogenesis procedure [14]: 75 µg of 7,12-dimethylbenz[a]anthracene (DMBA; Sigma-Aldrich, St. Louis, MO, USA) was applied topically to the shaved back skin of wild-type BALB/c mice (*n* = 18), followed by twice-weekly topical application of 10 µg of the tumour promoter phorbol 12-myristate 13-acetate (PMA, Sigma-Aldrich).

### Antibodies and treatment protocol

Control IgG and anti-IL-33 antibody (AF3626) were obtained from R&D Systems. For the in vivo experiments, the control antibody or the anti-IL-33 antibody were administered intraperitoneally once per week at a dose of 3 µg/mice starting 16 weeks after DMBA exposure [15]. The mice were euthanised 17 weeks after the first PMA cycle, and tumours were harvested for analysis.

### Isolation of leucocytes

After euthanising the mice, the skin and the tumour-draining lymph nodes were removed. Skin samples were processed as described previously [16]. Briefly, tumour samples were separated and placed in RPMI 1640 medium (Gibco, Grand Island, NY, USA) supplemented with 250 µg/mL collagenase (Boehringer Ingelheim Chemicals), 3000 U/mL DNase, 100 U/mL penicillin and 100 µg/ml streptomycin for 40 min at 37 °C. One cycle of cellular dissociation was performed for 4 min using a Medimachine (BD Biosciences). Cells were passed through a nylon mesh (30-µm pore size) and then processed for flow cytometry as described below. Tumour-draining lymph nodes were removed, and single-cell suspensions were obtained by passing the cells through a 70-µm cell strainer and prepared for flow cytometry.

### Flow cytometry

Surface and intracellular staining and flow cytometry were performed as described previously [14]. For intracellular staining, cells were fixed in 4% formaldehyde and then permeabilised using the Perm/Wash^™^ buffer kit (BD Biosciences), followed by incubation with antibodies. The following antibodies were used: anti-mouse CD19 (1D3), anti-mouse CD3 (145-2C11), anti-mouse CD4 PERCP (RM4-5), anti-mouse CD8 APC (53 − 6.7), anti-mouse IFNy PE (B27), anti-mouse IL-10 FITC (JES5-16E3) and anti-mouse IL-4-PE (11B11); the respective goat and rat isotype controls were used for each analysed antibody (BD Biosciences). Data were collected using an FACSCalibur (BD Immunocytometry Systems) and analysed using CellQuest software (BD Biosciences).

### Histological analysis

Haematoxylin and eosin–stained sections were reviewed by two pathologists to confirm the histopathological diagnosis. Formalin-fixed and paraffin-embedded samples were collected from each tumour specimen. All sections were analysed under an optical microscope, and microphotographs were collected using a digital camera (Leica DFC310 FX, Leica Microsystems GmbH, Wetzlar, Germany). Representative sections from each lesion were used for histopathological analysis.

### Statistical analysis

Statistical significance was determined with Student’s t-test when comparing two groups or one-way analysis of variance (ANOVA) when comparing three or more groups. The log-rank (Mantel–Cox) test was used to determine whether the anti-IL-33-treated mice had better outcomes than the wild-type mice. The data are presented as the mean ± standard error of the mean (SEM) or the mean ± standard deviation (SD). A P value ≤ 0.05 was considered statistically significant. The Prism 8.3 software program (GraphPad Software, San Diego, CA, USA) was used for statical analysis.

## Results

### IL-33 expression in human SCC lesions

**Fig. 1:**
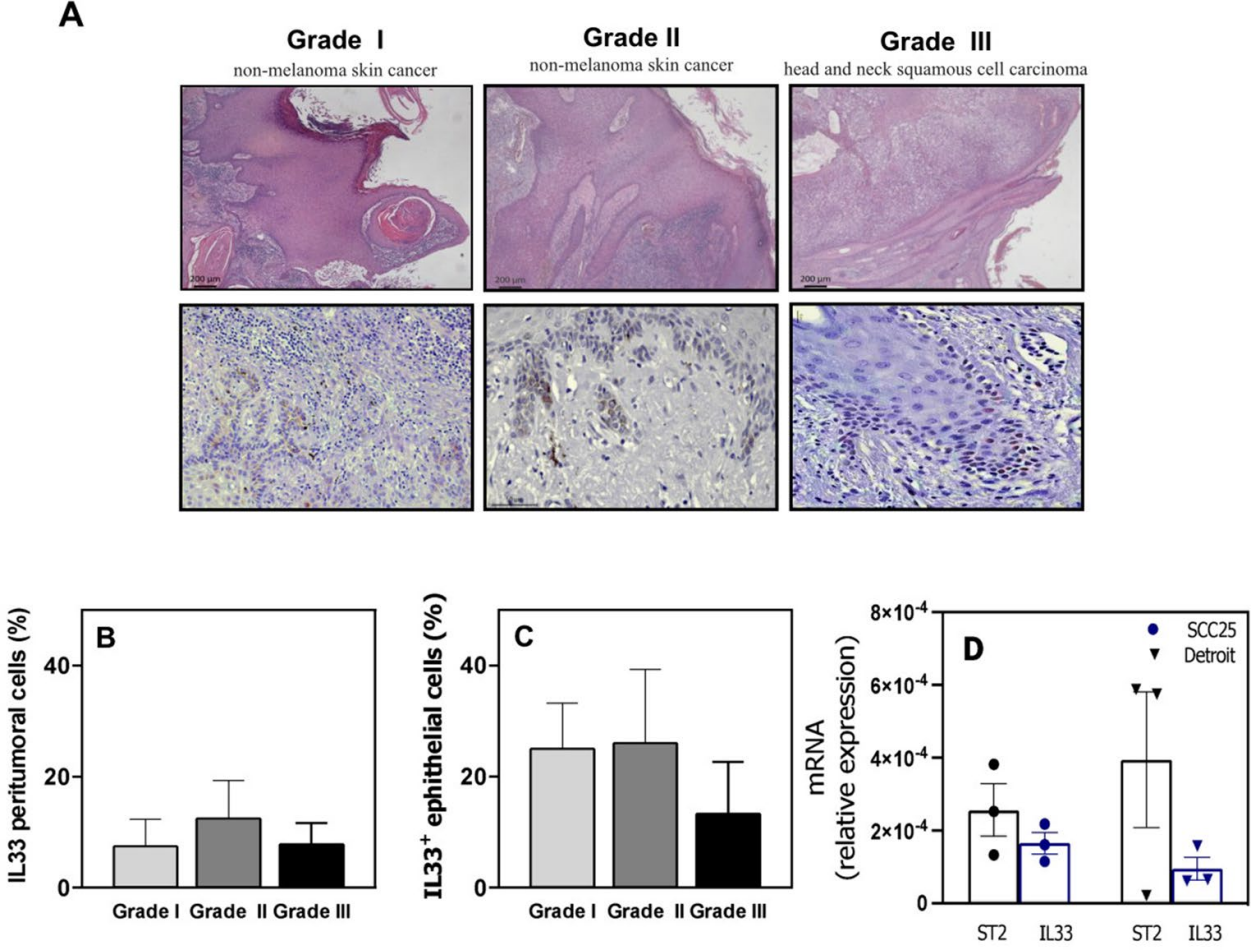
Interleukin 33 (IL-33) in squamous cell carcinoma (SCC). (**a**) Representative micrographs (scale bar = 200 μm) of three patients with SCC, including haematoxylin and eosin staining (first line) and immunohistochemical staining for IL-33 (second line). (**b**) The graph represents the mean ± standard deviation (SD) of the percentage of IL-33^+^ peritumoural cells. (**c**) The graph represents the mean ± SD of the number of IL-33^+^ epithelial cells. (**d**) Relative expression of *ST2* and *IL33* determined by quantitative polymerase chain reaction (mean ± standard error of the mean, *n* = 3) in SCC cell lines

IL-33 is associated with a mouse model of SCC development [10]; therefore, we investigated which cells may be the source of IL-33 in the tumour microenvironment in human SCC lesions. We analysed the IL-33 protein levels in paraffin-embedded tissue sections of SCC (Fig. 1). We detected IL-33 in the peritumoural stroma of nearly all tissue sections (Fig. 1a). The IL-33^+^ cells in SCC tissue sections showed typical cytoplasmic staining; they were epithelial and mononuclear cells (Fig. 1a). We detected IL-33^+^ cells with mononuclear cell morphology. Overall, there were fewer IL-33^+^ peritumoural cells than IL-33^+^ epithelial cells (Fig. 1b and c).

We also performed RT-qPCR to analyse *IL33* and *ST2* gene expression in two HNSCC cell lines. Detroit 562 cells were isolated from the pharynx of a patients with pharyngeal cancer and SCC-25 cells were isolated from the tongue of a patient with SCC. IL-33 and ST2 gene expression were not different between these two SCC lines (Fig. 1d). There were low IL-33 and ST2 mRNA levels in these cells.

### Effects of IL-33 on the motility, proliferation and invasiveness of SCC-25 and Detroit 562 cells

**Fig. 2:**
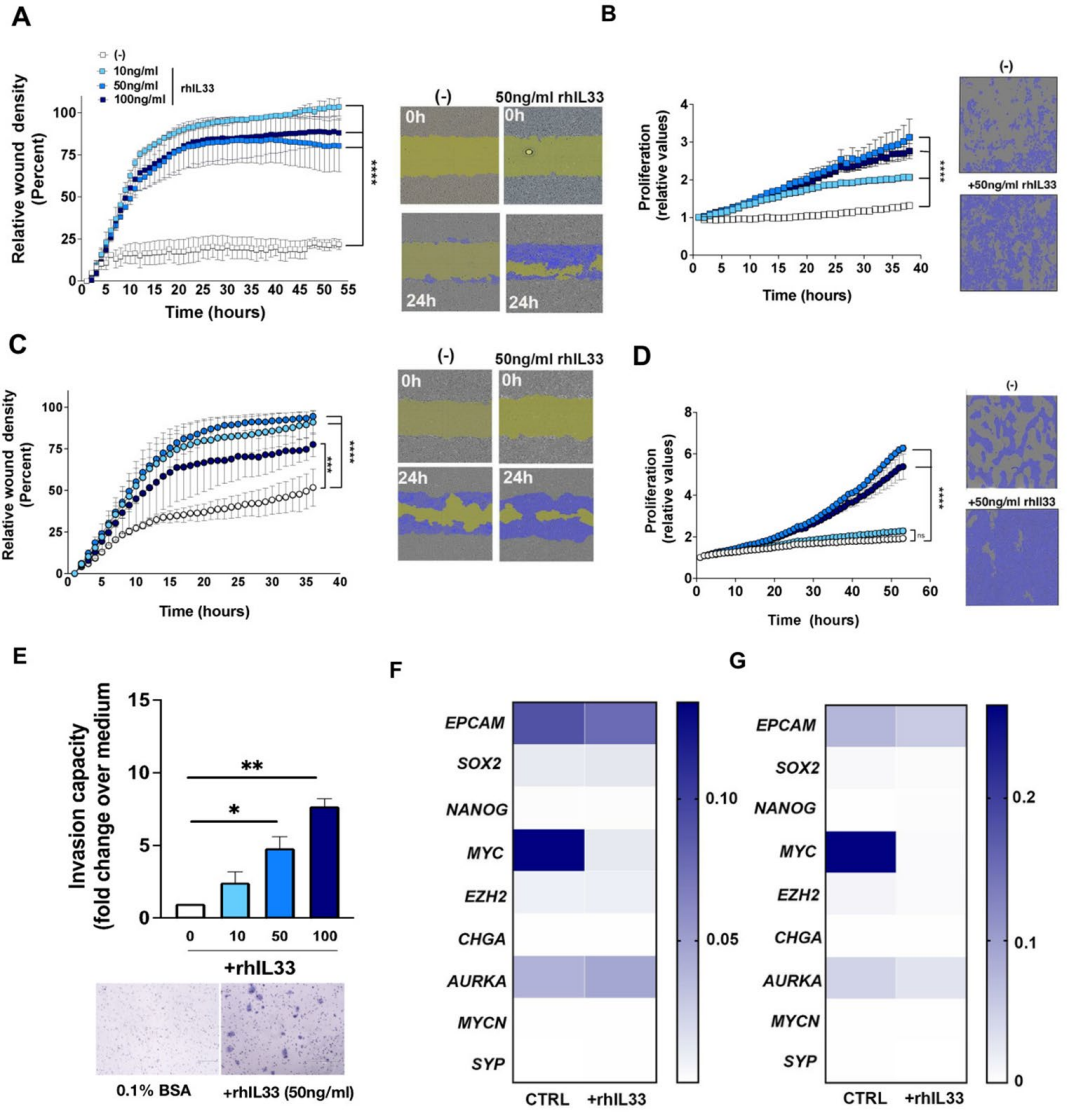
Effects of interleukin 33 (IL-33) in SCC25 and Detroit 562 cells. (**a**) Relative wound density curve of SCC25 cells for the IncuCyte analysis over 24 h. Representative images of scratch assays show scratches immediately after they were made (0 h) and after 24 h in the presence of IL-33 (right panels) versus positive control medium (left panels). The scale bars represent 300 μm. (**b**) Proliferation curve for SCC25 cells in the presence of IL-33. Representative images of the proliferation assay. (**c**) Relative wound density curve of Detroit 562 cells for the IncuCyte analysis over 24 h. Representative images of scratch assays show scratches immediately after they were made (0 h) and after 24 h in the presence of IL-33 (right panels) versus positive control medium (left panels). The scale bars represent 300 μm. (**d**) Proliferation curve for Detroit 562 cells in the presence of IL-33. Representative images of the proliferation assay. The data are shown as the mean ± standard error of the mean (SEM) from a single experiment and are representative of at least three experiments. (**e**) Transwell invasion assay for SCC25 cells at 48 h after incubation with IL-33 (10, 50 and 100 ng/ml). The data are shown as the mean ± SEM of cells counted in five representative microscopic fields per membrane and analysed using the ImageJ software. Representative images of invasion assay from two experiments. (**f**) Relative expression of *EPCAM*, *SOX2*, *NANOG*, *MYC*, *EZH2*, *CHGA*, *AURKA*, *MYCN* and *SYP* in SCC25 cells determined with quantitative polymerase chain reaction (mean ± SEM, *n* = 3). (**g**) Relative expression of *EPCAM*, *SOX2*, *NANOG*, *MYC*, *EZH2*, *CHGA*, *AURKA*, *MYCN* and *SYP* in Detroit 562 cells determined with quantitative polymerase chain reaction (mean ± SEM, *n* = 3). *P < 0.05, (**P < 0.01), ***P < 0.001 and ****P < 0.0001

IL-33 has a well-known impact on immune cells, especially Th2 cells in allergic disease and parasitic infections. However, in cancer immunity IL-33 can display both pro- and anti-tumoural functions, depending on the specific microenvironment. We evaluated the effect of IL-33 on the biology of SCC-25 and Detroit 562 cells, including their motility and growth. Our initial analysis with IncuCyte technology showed a significant dose-dependent effect of rhIL-33 on the migratory capacity of SCC-25 cells. Compared with the control, the migratory capacity of SCC-25 cells increased by 92.6% ± 3.4% (10 ng/mL rhIL-33), 80.7% ± 22.5% (50 ng/mL rhIL-33) and 83.0% ± 23.2% (100 ng/mL rhIL-33) (Fig. 2a and b). rhIL-33 treatment of SCC-25 cells also induced a significant dose-dependent increase in proliferation (Fig. 2b); 50 ng/mL rhIL-33 produced the maximum effect. We next analysed the effect of IL-33 on the biology of Detroit 562 cells, which are cells derived from metastatic sites [17]. We observed partial motility of Detroit 562 cells independent of the presence of rhIL-33 (Fig. 2c). Both 50 and 100 ng/mL rhIL-33 induced the proliferation of Detroit 562 cells in a similar manner. However, 20 ng/mL rhIL-33 could not induce a increase in proliferation (Fig. 2d).

Next, we asked whether IL-33 modulates the invasiveness of SCC cell lines. We stimulated SCC-25 cells with different IL-33 concentrations in a transwell assay. There was a dose-dependent increase in the invasive capacity (Fig. 2e). Compared with control (0 ng/mL rhIL-33, 1% invasive capacity), the invasive capacity increased to 2.43-fold ± 1.03-fold (10 ng/mL rhIL-33), 4.8-fold ± 1.13-fold (50 ng/mL rhIL-33) and 7.7-fold ± 0.70-fold (100 ng/mL rhIL-33). These results indicate a direct effect on the biology of SCC-25 cells based on their migratory, proliferative and invasive capacities, suggesting that IL-33 induces a tumour-promoting phenotype in SCC-25 cells. Although Detroit 562 cells are derived from a metastatic site of SCC, their invasive capacity in both Matrigel and collagen transwell assays was remarkably absent or much lower than that of SCC-25 cells (data do not show).

The cellular plasticity of cancer is characterised by functional and phenotypic changes between mesenchymal and epithelial states; it correlates with invasion, proliferation and, consequently underlies tumour heterogeneity and progression [12, 16]. We found that rhIL-33 increased the biological functions on SCC-25 cells – and to some extent in Detroit 562 cells – that are associated with aggressiveness. These findings led us to investigate whether cellular plasticity may be involved in this biological phenomenon. We analysed the expression of some genes related to EMT (*EPCAM* and *SOX2*), stemness (*NANOG*, *MYC* and *EZH2*) and neuroendocrine differentiation (*CHGA*, *AURKA*, *MYCN* and *SYP*). We found that SCC-25 and Detroit 562 cells do not express most of these markers, except for *EPCAM*, *SOX2*, *MYC*, *EZH2* and *AURKA*. Of these genes, *MYC* showed the highest expression. Surprisingly, rhIL-33 significantly downregulated *MYC* expression (Fig. 2f and g). These data suggest that the molecular mechanism by which rhIL-33 induces a tumour-promoting phenotype in SCC-25 cells is independent of EMT, stemness and neuroendocrine differentiation.

### Effects of IL-33 inhibition on SCC and T cell activation

**Fig. 3:**
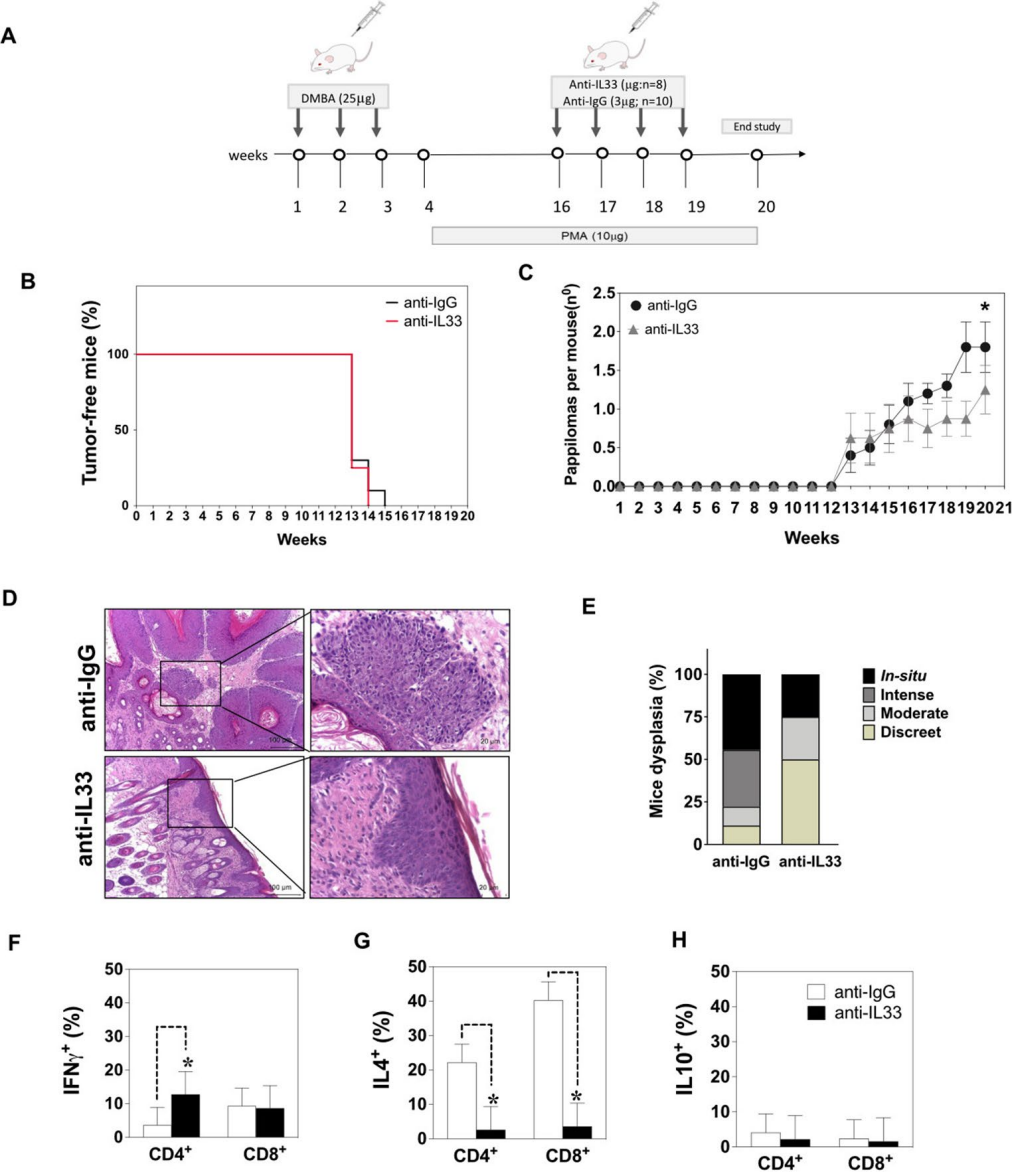
Interleukin 33 (IL-33) blockade decreases the development of squamous cell carcinoma. (**a**) Schematic representation of the skin chemical carcinogenesis protocol. (**b**) Kaplan–Meier curve showing the tumour-free mice. (c) The number of papilloma-like lesions per mouse. (**d**) Representative pictures of lesion growth patterns from anti-IL-33-treated mice and IgG-treated-mice (10× and 20×). (**e**) Haematoxylin and eosin–stained sections were scored to grade their level of dysplasia. The scale bars represent 50 µM. (f–h) Percentage of IFNγ^+^, IL-4^+^ and IL-10^+^ cells in lymph nodes from anti-IL-33-treated mice and IgG-treated-mice. The bars represent the mean ± standard deviation. *P < 0.05

We next investigated whether treatment of tumour-bearing mice with a neutralising anti-mouse IL-33 antibody (3 µg/mouse delivered once a week) had an impact on tumour growth and the immune compartment (Fig. 3a). IL-33 blockade significantly reduced the disease burden compared with mice treated with the IgG control antibody (*P* < 0.05; Fig. 3b and c). However, the mortality rate was not different between anti-IL-33-treated and IgG-treated mice. We confirmed these findings histologically (Fig. 3d). Histological evaluation of the skin tumours showed that 50% of the anti-IL-33-treated mice exhibited mild hyperplasia (grade I) (Fig. 3e), while only 25% were classified as SCC in situ (Fig. 3e). In comparison, the wild-type mice exhibited high-grade hyperplasia and dysplasia.

We next investigated whether IL-33 blockade modulates the immune compartment in tumour-draining lymph nodes. The percentage of CD4^+^IFNγ^+^ T cells present in these lymph nodes was significantly higher in anti-IL-33-treated mice (12.8%) compared with IgG-treated mice (3.6%) (Fig. 3f). The percentage of CD8^+^IFNγ^+^ T cells was similar between the groups, 9.3% in anti-IL-33-treated mice and 9.6% in IgG-treated mice (Fig. 3f). The intracellular IL-4 expression by CD4 ^+^ T cells was significantly decreased in anti-IL-33-treated mice (2.6%) compared with IgG-treated mice (22.2%) (Fig. 3g). There were no significant differences in the percentage of CD4^+^IL-10^+^ and CD8^+^IL-10^+^ T cells (Fig. 3h). These results suggest that blocking IL-33 leads to a modulation of the immune compartment to a pro-inflammatory phenotype mediated by CD4^+^ T cells.

## Discussion

IL-33 exhibits pleiotropic functions in inflammatory diseases and particularly in cancer. This cytokine plays a dual role in tumourigenesis that depends on the tumour microenvironment, including the cellular interactions and the inflammatory context [18]. Our group previously demonstrated the increased susceptibility of ST2-deficient mice to SCC progression [10], raising the intriguing possibility that IL-33 could be exploited for SCC treatment. In the current study, we investigate the role of IL-33 in two epithelial HNSCC cell lines as well as in the immune compartment. Blocking IL-33 in vivo resulted in a skewing of the Th1 immune profile and, consequently, a reduction in the tumour burden after SCC development. The presence of IL-33 also exerted biological alterations directly on the tumour cells, leading to increased motility and proliferation.

IL-33 is expressed by several cell types within the tumour microenvironment and exhibits different or even opposite functions under varying circumstances [19, 20]. IL-33 knockdown decreases the metastasis and invasiveness of oesophageal SCC cell lines (KYSE-450 and Eca-109), whereas IL-33 overexpression shows the opposite effect. However, another study reported that tumoural expression of IL-33 inhibits tumour growth and modifies the tumour microenvironment, maintaining the controversy about the functional role of IL-33 in cancer. In addition, IL-33 can act directly on tumour cells to enhance chemoresistance [21]. In the present study, we showed that IL-33 is expressed in SCC tissue samples and that IL-33 treatment significantly enhanced the migration and proliferation of SCC-25 and Detroit 562 cells.

In HNSCC, IL-33 has a heterogeneous role among tumours of different sites. IL-33 has been detected in cancer-associated fibroblasts (CAFs) and is a critical mediator in CAF-induced invasiveness [22]. Another study suggested that stroma- and epithelium-derived IL-33 produce different responses [23]. In laryngeal squamous cell carcinoma (LSCC), IL-33 can act as a pro-tumour factor. In oral cavity squamous cell carcinoma (OCSCC), IL-33 may act as an anti-tumour factor. In oropharyngeal squamous cell carcinoma (OPSCC), the role of IL-33 has not yet been determined. IL-33 in LSCC is primarily derived from endothelial cells, whereas IL-33 in OCSCC is primarily derived from endothelial and epithelial cells [23]. The effect of IL-33 on the invasiveness of SCC cells has also been studied. It acts as a chemoattractant cytokine, increasing the invasiveness of SCC25 cells. Supporting these data, studies involving HNSCC, pancreatic carcinoma and colon carcinoma cell lines suggest that IL-33 is an autocrine/paracrine mediator of carcinoma cell invasiveness and metastasis [22–25]. In a mouse model of SCC, IL-33 from CAFs regulated the invasiveness of FADU cells through paracrine mechanisms [22]. By contrast, our data demonstrated that different IL-33 concentrations had no effects on the invasiveness of Detroit 562 cells. Overall, there are controversial data regarding the use of different SCC cell lines, their passages and tissue origin, and these factors could have an impact on the outcome of these experiments. The motility of both SCC cell lines was significantly increased in the presence of IL-33. Similarly, keratinocytes isolated from IL-33-knockout mice or pre-designed small interfering RNA (siRNA), which effectively knocks down human cellular IL-33 in keratinocytes, showed delayed scratch wound closure in vitro [26]. It has been reported that ILC2s exert an essential role in the tumour-driven IL-33/ST2/IL-13 axis by promoting the migration and invasion of colorectal cancer cells, which are key factors promoting metastasis [27]. PPARγ is key in this interaction between ILC2s and cancer cells: it supports the pro-tumoural functions of ILC2s, and this function might be conserved across different IL-33-dependent tumours [28]. Future studies are required to establish the downstream pathway contribution of IL-33-driven SCC cell motility and proliferation.

It is now clear that IL-33 and cancer cell interactions within the tumour microenvironment regulate cancer stem cell properties and play an important role in tumour progression and metastasis [29]. Recently, researchers reported that IL-33 could induce EMT and promote oesophageal SCC metastasis [20, 30]. We found decreased EMT-related gene expression, including *MYC* (encodes c-MYC), in IL-33-treated SCC cells. *MYC* is a proto-oncogene that is constitutively and aberrantly expressed in over 70% of human cancers. In oesophageal SCC tissues, c-MYC levels are significantly higher than noncancerous tissues [31]. This transcription factor has functional importance in the orchestration of transcription pathways that regulate cell cycle progression, metabolism and survival [32], and is directly associated with metastasis [33]. c-MYC is also responsible for the crosstalk between breast cancer cells and the tumour microenvironment by regulating proteins in cancer cells as well as immune infiltration [34]. Inhibition of c-MYC expression increases the apoptosis of SCC cells [35]. In our study, the presence of IL-33 resulted in significant *MYC* downregulation in both HNSCC cell lines. We also demonstrated that exogenous IL-33 increased SCC cell proliferation in vitro, indicating that IL-33 may play a role in cell proliferation independent of c-MYC signalling. *MYC* depletion in colorectal cancer cell lines leads to cell cycle arrest by altering p53 signalling and its downstream effectors [36]. *MYC* gene expression is closely regulated at the transcriptional and post-transcriptional levels, and c-MYC activity is controlled via post-translational modifications [37]. Further, transcript levels by themselves are not necessarily sufficient to predict protein levels and thus explain genotype–phenotype relationships [38]. Looking downstream of c-MYC activation will provide a better understanding of the biological processes induced by IL-33 in SCC. One limitation of this study is that we looked at *MYC* expression at one time point. In future studies we need to examine additional time points to fully understand the transcriptional regulation of this protein.

The IL-33/ST2 axis plays an important immunomodulating in the tumour microenvironment, where it acts on immune cell populations associated with type 2 and regulatory immunity. In particular, IL-33 directly activates ST2-expressing ILC2s and induces type 2 cytokine secretion [39]. In cancer immunity, this axis can display both pro- and anti-tumoural functions, depending on the specific microenvironment, that is, crosstalk between stroma and immune cells. We aimed to determine how endogenous IL-33 affects T cell–dependent responses in an in vivo model of SCC development. In our study, mice subjected to IL-33 neutralisation exhibited less tumour growth. Likewise, IL-33 neutralisation also decreased the size and number of tumours in an experimental model of colorectal cancer [40]. Similarly, anti-IL-33 antibody treatment has been shown to inhibit glioblastoma and ovarian cancer in experimental models [41–43]. In another study, anti-IL-33 antibody treatment reduced inflammation and improved the efficacy of chemotherapy in a colorectal cancer model [44]. In our study, anti-IL-33 antibody treatment reduced dysplasia, supporting the hypothesis that the presence of IL-33 in the tumour microenvironment is correlated with faster and more aggressive tumour development in some tumour models [45]. In preclinical trials, blocking IL-33 signalling with an anti-IL-33 or anti-ST2 antibody showed efficacy in patients with lung cancer [46, 47].

Analysis of the immune response in tumour-draining lymph nodes showed that the percentage of CD4^+^IFNγ^+^ cells was significantly higher in anti-IL-33 treated mice. These findings clearly indicate that IL-33 modulates CD4^+^IFNγ^+^ cell activation, suggesting that anti-IL-33 treatment in SCC stimulates the differentiation of CD4^+^IFNγ^+^ T cells and inhibits the activation of IL-4-producing CD4^+^ cells, thus favouring an anti-tumour protective immune response. CD4^+^IL-4^+^ T lymphocytes act as indirect promoters of invasion and metastasis by regulating the increase in macrophages in the tumour microenvironment [47]. Blocking IL-33 may influence the cell activation profile and recruitment to the tumour microenvironment. Despite the development of a predominantly Th1 immune response in the lymph nodes, the same cell migration could not have occurred given the profile of chemokines produced in the tumour microenvironment. In addition, activated and differentiated CD4^+^ T cells in lymph nodes can present modulated profiles in an inflammatory microenvironment [48, 49] In the current study, we found that blocking IL-33 in vivo significantly decreased the inflammatory cell infiltration in tumour samples and significantly decreased the number of CD4^+^ T cells, dendritic cells and macrophages (Supplementary Fig. 1). These results suggest that anti-IL-33 antibody treatment may impact the development of immune responses during SCC development. These findings are consistent with a study of non-small cell lung cancer in mice [45]. After treating immunodeficient mice with an anti-IL-33 antibody, cancer growth, M2 macrophage polarisation and the number of Tregs at the tumour site were reduced. These findings suggest the blocking IL-33 could be a therapeutic option for patients with non-small cell lung cancer [46].

**Fig. 4:**
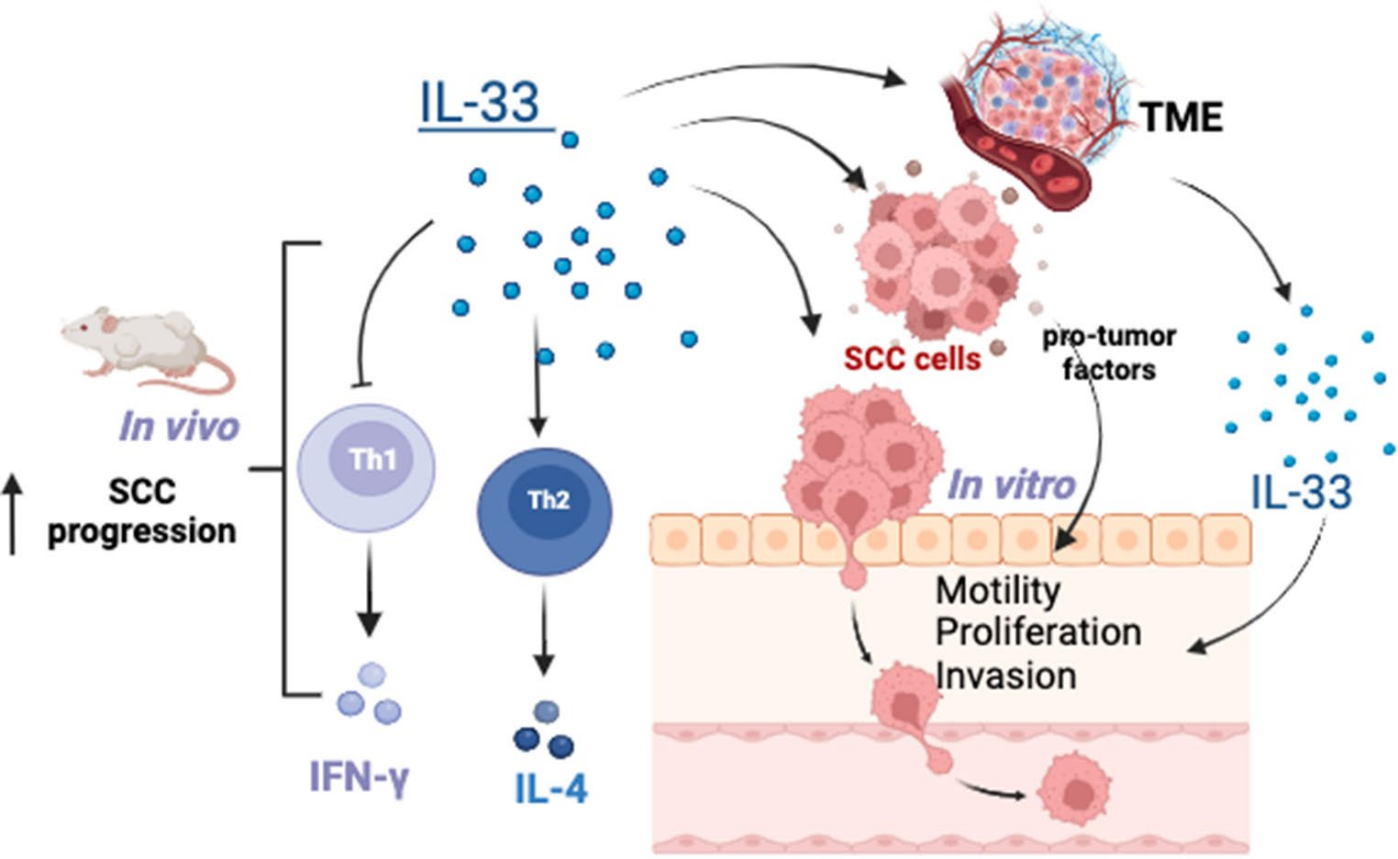
Proposed model of the role of interleukin 33 (IL-33) in squamous cell carcinoma (SCC) growth via a dual effect on tumour proliferation, migration and invasion, and T cell activation. IL-33 + epithelial and mononuclear cells are found in in the SCC lesions (Fig. 1). The release of this cytokine induces changes in the biology of SCC cells by promoting a pro-tumour phenotype directly (Fig. 2) or indirectly by inducing other factors in the tumour microenvironment (not tested). The neutralisation of IL-33 restricts SCC outgrowth, decreases Th2-derived IL-4 and increases accumulation of Th1 IFNγ-producing cells in tumour-draining lymph nodes (Fig. 3), and thus represents an effective and promising strategy for SCC treatment. Created with Biorender

In conclusion, IL-33 supports tumour growth and influences immune surveillance in the tumour microenvironment, favouring immune escape of tumour cells. We found that IL-33 in the SCC biopsies was mostly expressed in epithelial and mononuclear cells (Fig. 4). IL-33 blockade restricted SCC outgrowth, decreased Th2-derived IL-4 and increased the accumulation of Th1 IFNγ-producing cells in tumour-draining lymph nodes, and thus represents a promising SCC treatment strategy (Fig. 4). These findings provide new insight into SCC pathogenesis. Future studies are necessary to understand the mechanisms by which IL-33 is a pro-tumour cytokine during SCC and provide insight for developing novel therapeutics against SCC in clinical practice.

### Electronic supplementary material

Below is the link to the electronic supplementary material.

Supplementary Material 1

## References

[1] Sánchez-Danés A, Blanpain C (2018). Deciphering the cells of origin of squamous cell carcinomas. Nat Rev Cancer.

[2] Palmieri V, Ebel JF, Ngo Thi Phuong N, Klopfleisch R, Vu VP, Adamczyk A, Zöller J, Riedel C, Buer J, Krebs P, Hansen W, Pastille E, Westendorf AM (2021). Interleukin-33 signaling exacerbates experimental infectious colitis by enhancing gut permeability and inhibiting protective Th17 immunity. Mucosal Immunol.

[3] Xiao P, Wan X, Cui B, Liu Y, Qiu C, Rong J, Zheng M, Song Y, Chen L, He J, Tan Q, Wang X, Shao X, Liu Y, Cao X, Wang Q (2015). Interleukin 33 in tumor microenvironment is crucial for the accumulation and function of myeloid-derived suppressor cells. Oncoimmunology.

[4] Wasmer MH, Krebs P (2017). The role of IL-33-Dependent inflammation in the Tumor Microenvironment. Front Immunol.

[5] Fournié JJ, Poupot M (2018). The pro-tumorigenic IL-33 involved in Antitumor Immunity: a Yin and Yang Cytokine. Front Immunol.

[6] Pastille E, Wasmer MH, Adamczyk A, Vu VP, Mager LF, Phuong NNT, Palmieri V, Simillion C, Hansen W, Kasper S, Schuler M, Muggli B, McCoy KD, Buer J, Zlobec I, Westendorf AM, Krebs P (2019). The IL-33/ST2 pathway shapes the regulatory T cell phenotype to promote intestinal cancer. Mucosal Immunol.

[7] Shen JX, Liu J, Zhang GJ (2018). Interleukin-33 in malignancies: friends or foes?. Front Immunol.

[8] Gao X, Wang X, Yang Q, Zhao X, Wen W, Li G, Lu J, Qin W, Qi Y, Xie F, Jiang J, Wu C, Zhang X, Chen X, Turnquist H, Zhu Y, Lu B (2015) Tumoral expression of IL-33 inhibts tumor growth and modifies the tumor microenvironment through CD8 + T and NK cells. Journal of immunology (Baltimore, Md.: 1950), 194(1), 438–445. 10.4049/jimmunol.1401344 10.4049/jimmunol.1401344 PMC4272901 25429071

[9] Kim J, Kim W, Moon UJ, Kim HJ, Choi HJ, Sin JI, Park NH, Cho HR, Kwon B (2016). Intratumorally establishing type 2 innate lymphoid cells blocks Tumor Growth. J Immunol (Baltimore Md: 1950).

[10] Amôr NG, de Oliveira CE, Gasparoto TH, Vilas Boas VG, Perri G, Kaneno R, Lara VS, Garlet GP, da Silva JS, Martins GA, Hogaboam C, Cavassani KA, Campanelli AP (2018). ST2/IL-33 signaling promotes malignant development of experimental squamous cell carcinoma by decreasing NK cells cytotoxicity and modulating the intratumoral cell infiltrate. Oncotarget.

[11] Taniguchi S, Elhance A, Van Duzer A, Kumar S, Leitenberger JJ, Oshimori N (2020) Tumorinitiating cells establish an IL-33-TGF-β niche signaling loop to promote cancer progression, vol 369. Science (New York, p eaay1813. 6501) 10.1126/science.aay1813 10.1126/science.aay1813 PMC10870826 32675345

[12] Fang M, Li Y, Huang K, Qi S, Zhang J, Zgodzinski W, Majewski M, Wallner G, Gozdz S, Macek P, Kowalik A, Pasiarski M, Grywalska E, Vatan L, Nagarsheth N, Li W, Zhao L, Kryczek I, Wang G, Wang Z, Wang L (2017). IL33 promotes Colon Cancer Cell Stemness via JNK activation and macrophage recruitment. Cancer Res.

[13] Cavassani KA, Meza RJ, Habiel DM, Chen JF, Montes A, Tripathi M, Martins GA, Crother TR, You S, Hogaboam CM, Bhowmick N, Posadas EM (2018). Circulating monocytes from prostate cancer patients promote invasion and motility of epithelial cells. Cancer Med.

[14] Ramos RN, Oliveira CE, Gasparoto TH, Malaspina TS, Belai EB, Cavassani KA, Garlet GP, Silva JS, Campanelli AP (2012). CD25 + T cell depletion impairs murine squamous cell carcinoma development via modulation of antitumor immune responses. Carcinogenesis.

[15] Dominguez D, Ye C, Geng Z, Chen S, Fan J, Qin L, Long A, Wang L, Zhang Z, Zhang Y, Fang D, Kuzel TM, Zhang B (2017) Exogenous IL-33 Restores Dendritic Cell Activation and Maturation in Established Cancer. Journal of immunology (Baltimore, Md.: 1950), 198(3), 1365–1375. 10.4049/jimmunol.1501399 10.4049/jimmunol.1501399 PMC5263113 28011934

[16] Teeuwssen M, Fodde R (2019). Cell heterogeneity and phenotypic plasticity in metastasis formation: the case of Colon cancer. Cancers.

[17] 17.Sano D, Xie TX, Ow TJ, Zhao M, Pickering CR, Zhou G, Sandulache VC, Wheeler DA, Gibbs RA, Caulin C, Myers JN (2011) Disruptive TP53 mutation is associated with aggressive disease characteristics in an orthotopic murine model of oral tongue cancer. Clin cancer Research: Official J Am Association Cancer Res 17(21):6658–6670. 10.1158/1078-0432.CCR-11-0046 10.1158/1078-0432.CCR-11-0046 PMC3207013 21903770

[18] Yeoh WJ, Vu VP, Krebs P (2022). IL-33 biology in cancer: an update and future perspectives. Cytokine.

[19] Gao X, Wang X, Yang Q, Zhao X, Wen W, Li G, Lu J, Qin W, Qi Y, Xie F, Jiang J, Wu C, Zhang X, Chen X, Turnquist H, Zhu Y, Lu B (2015). Tumoral expression of IL-33 inhibits tumor growth and modifies the tumor microenvironment through CD8 + T and NK cells. J Immunol (Baltimore Md: 1950).

[20] Yue Y, Lian J, Wang T, Luo C, Yuan Y, Qin G, Zhang B, Zhang Y (2020). Interleukin-33-nuclear factor-κB-CCL2 signaling pathway promotes progression of esophageal squamous cell carcinoma by directing regulatory T cells. Cancer Sci.

[21] Larsen KM, Minaya MK, Vaish V, Peña MMO (2018). The role of IL-33/ST2 pathway in Tumorigenesis. Int J Mol Sci.

[22] Chen SF, Nieh S, Jao SW, Wu MZ, Liu CL, Chang YC, Lin YS (2013). The paracrine effect of cancer-associated fibroblast-induced interleukin-33 regulates the invasiveness of head and neck squamous cell carcinoma. J Pathol.

[23] Peng L, Sun W, Chen L, Wen WP (2021). The role of Interleukin-33 in Head and Neck squamous cell carcinoma is determined by its Cellular sources in the Tumor Microenvironment. Front Oncol.

[24] Liu X, Zhu L, Lu X, Bian H, Wu X, Yang W, Qin Q (2014). IL-33/ST2 pathway contributes to metastasis of human colorectal cancer. Biochem Biophys Res Commun.

[25] Schmieder A, Multhoff G, Radons J (2012). Interleukin-33 acts as a pro-inflammatory cytokine and modulates its receptor gene expression in highly metastatic human pancreatic carcinoma cells. Cytokine.

[26] Tsuda H, Tominaga SI, Ohtsuki M, Komine M (2022). Nuclear IL-33 regulates cytokinesis and cell motility in normal human epidermal keratinocytes. J Dermatol Sci.

[27] Pijuan J, Barceló C, Moreno DF, Maiques O, Sisó P, Marti RM, Macià A, Panosa A (2019). In vitro cell Migration, Invasion, and adhesion assays: from cell imaging to Data Analysis. Front cell Dev Biology.

[28] Ercolano G, Gomez-Cadena A, Dumauthioz N, Vanoni G, Kreutzfeldt M, Wyss T, Michalik L, Loyon R, Ianaro A, Ho PC, Borg C, Kopf M, Merkler D, Krebs P, Romero P, Trabanelli S, Jandus C (2021). PPARɣ drives IL-33-dependent ILC2 pro-tumoral functions. Nat Commun.

[29] Nallasamy P, Nimmakayala RK, Parte S, Are AC, Batra SK, Ponnusamy MP (2022). Tumor microenvironment enriches the stemness features: the architectural event of therapy resistance and metastasis. Mol Cancer.

[30] Bhat AA, Nisar S, Maacha S, Carneiro-Lobo TC, Akhtar S, Siveen KS, Wani NA, Rizwan A, Bagga P, Singh M, Reddy R, Uddin S, Grivel JC, Chand G, Frenneaux MP, Siddiqi MA, Bedognetti D, El-Rifai W, Macha MA, Haris M (2021). Cytokine-chemokine network driven metastasis in esophageal cancer; promising avenue for targeted therapy. Mol Cancer.

[31] Xin Z, Xin G, Shi M, Song L, Wang Q, Jiang B, Liu X (2018). Inhibition of MUC1-C entering nuclear suppresses MYC expression and attenuates malignant growth in esophageal squamous cell carcinoma. OncoTargets Therapy.

[32] Dang CV (2013). MYC, metabolism, cell growth, and tumorigenesis. Cold Spring Harbor Perspect Med.

[33] Kubokura H, Tenjin T, Akiyama H, Koizumi K, Nishimura H, Yamamoto M, Tanaka S (2001). Relations of the c-myc gene and chromosome 8 in non-small cell lung cancer: analysis by fluorescence in situ hybridization. Annals Thorac Cardiovasc Surgery: Official J Association Thorac Cardiovasc Surg Asia.

[34] Gao FY, Li XT, Xu K, Wang RT, Guan XX (2023). c-MYC mediates the crosstalk between breast cancer cells and tumor microenvironment. Cell Communication Signaling: CCS.

[35] Zhao S, An L, Yang X, Wei Z, Zhang H, Wang Y (2022). Identification and validation of the role of c-Myc in head and neck squamous cell carcinoma. Front Oncol.

[36] Hermeking H, Funk JO, Reichert M, Ellwart JW, Eick D (1995). Abrogation of p53-induced cell cycle arrest by c-Myc: evidence for an inhibitor of p21WAF1/CIP1/SDI1. Oncogene.

[37] Spiniello M, Steinbrink MI, Cesnik AJ, Miller RM, Scalf M, Shortreed MR, Smith LM 2019 Comprehensive in vivo identification of the c-Myc mRNA protein interactome using HyPR-MS. RNA (New York, N.Y.), 25(10), 1337–1352. 10.1261/rna.072157.119 10.1261/rna.072157.119 PMC6800478 31296583

[38] Liu Y, Beyer A, Aebersold R (2016). On the Dependency of Cellular protein levels on mRNA abundance. Cell.

[39] Takatori H, Makita S, Ito T, Matsuki A, Nakajima H (2018) Regulatory Mechanisms of IL-33-ST2-Mediated Allergic Inflammation. Frontiers in immunology, 9, 2004. 10.3389/fimmu.2018.02004 10.3389/fimmu.2018.02004 PMC6131616 30233590

[40] Tanaka J, Irié T, Yamamoto G, Yasuhara R, Isobe T, Hokazono C, Tachikawa T, Kohno Y, Mishima K (2015). ANGPTL4 regulates the metastatic potential of oral squamous cell carcinoma. J oral Pathol Medicine: Official Publication Int Association Oral Pathologists Am Acad Oral Pathol.

[41] Zhang JF, TaoT, Wang K, Zhang GX, Yan Y, Lin HR, Li Y, Guan MW, Yu JJ, Wang XD (2019). IL33/ST2 axis promotes glioblastoma cell invasion by accumulating tenascin-C. Sci Rep.

[42] Wang L, Hu J, Qiu D, Gao H, Zhao W, Huang Y, Jiang T, Zhou J, Chen Y (2019). Dual-specificity phosphatase 5 suppresses ovarian cancer progression by inhibiting IL-33 signaling. Am J Translational Res.

[43] Nagaoka S, Yamada D, Eguchi H, Yokota Y, Iwagami Y, Asaoka T, Noda T, Kawamoto K, Gotoh K, Kobayashi S, Miyoshi E, Doki Y, Mori M (2021). The blockade of interleukin-33 released by hepatectomy would be a promising treatment option for cholangiocarcinoma. Cancer Sci.

[44] Guabiraba R, Besnard AG, Menezes GB, Secher T, Jabir MS, Amaral SS, Braun H, Lima-Junior RC, Ribeiro RA, Cunha FQ, Teixeira MM, Beyaert R, Graham GJ, Liew Y (2014). IL-33 targeting attenuates intestinal mucositis and enhances effective tumor chemotherapy in mice. Mucosal Immunol.

[45] Akimoto M, Maruyama R, Takamaru H, Ochiya T, Takenaga K (2016). Soluble IL-33 receptor sST2 inhibits colorectal cancer malignant growth by modifying the tumour microenvironment. Nat Commun.

[46] Wang K, Shan S, Yang Z, Gu X, Wang Y, Wang C, Ren T (2017). IL-33 blockade suppresses tumor growth of human lung cancer through direct and indirect pathways in a preclinical model. Oncotarget.

[47] Yang K, Tian C, Zhang C, Xiang M (2022). The controversial role of IL-33 in Lung Cancer. Front Immunol.

[48] DeNardo DG, Barreto JB, Andreu P, Vasquez L, Tawfik D, Kolhatkar N, Coussens LM (2009). CD4(+) T cells regulate pulmonary metastasis of mammary carcinomas by enhancing protumor properties of macrophages. Cancer Cell.

[49] Sallusto F (2016). Heterogeneity of human CD4(+) T cells against microbes. Annu Rev Immunol.

