# Supplementary figures and images for: Interleukin 33 supports squamous cell carcinoma growth via a dual effect on tumour proliferation, migration and invasion, and T cell activation
Source: Cancer Immunol Immunother. 2024 Apr 25;73(6):110. doi: 10.1007/s00262-024-03676-8 (PMC11045681; doi:10.1007/s00262-024-03676-8)

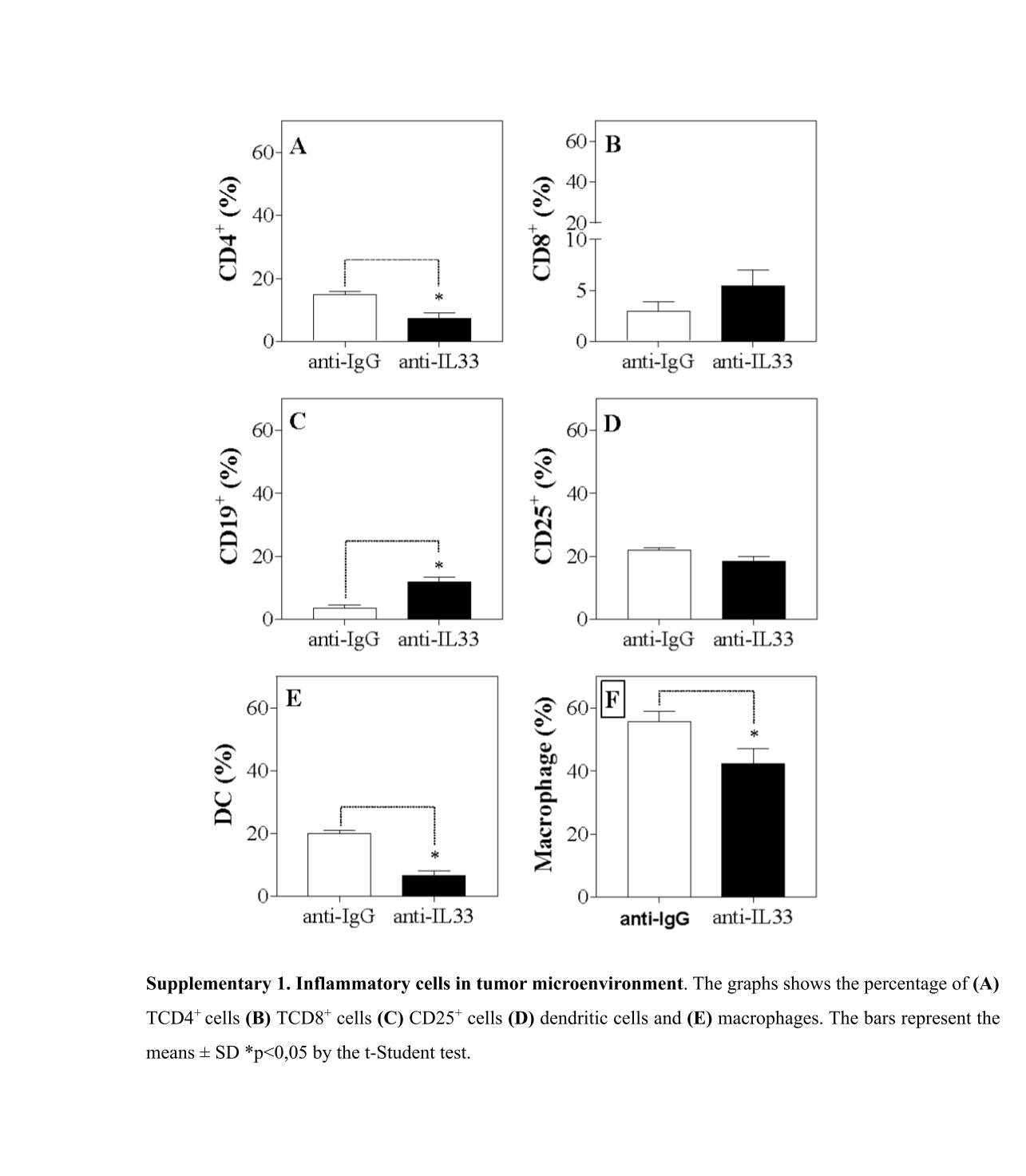

Supplement: Supplementary file 1 — Supplementary Material 1 [file 262_2024_3676_MOESM1_ESM.jpg]
